# Chemical Mixtures: Hayes Responds

**Published:** 2006-09

**Authors:** Tyrone B. Hayes

**Affiliations:** Department of Integrative Biology, University of California, Berkeley, California, E-mail: tyrone@berkeley.edu

LeBlanc and Wang point out that we did not demonstrate synergy; they stated that “to invoke synergy, one must—at a minimum—exclude the possibility of concentration or response additivity.” In fact, we did not “invoke synergy”: in our article, not once did we use the word “synergy.” LeBlanc and Wang themselves “invoke synergy” simply to show that it cannot be invoked from the current study; their problem is with what they inferred from our article, not with any claims that we made.

LeBlanc and Wang also point out that our “experiments were not designed to assess concentration additivity, so no judgment can be made either in favor of or against the possibility that the toxicity of the mixture represented concentration additivity.” We agree. This was not the aim of the study. In fact, LeBlanc and Wang’s thesis here is merely a restatement of our own conclusions:

The present effects of mixtures cannot be assigned to the categories of concentration additive or response additive. (Hayes et al. 2006; p. 47)

and

The examinations needed to characterize pesticide interactions as concentration additive or response additive … are difficult to design and carry out and present new challenges to regulators. Such studies are necessary, however (Hayes et al. 2006; p. 48)

Finally, LeBlanc and Wang examined our data for response additivity using a simple model and testing select parameters that fit their model while ignoring others. In our study we examined effects of nine pesticides alone (0.1 ppb) or in three different mixtures at 0.1 ppb and 10 ppb on leopard frog (*Rana pipiens*) larvae. Each treatment (30 larvae/tank) was replicated three times (1,350 larvae total). We assessed effects on 10 parameters: time to foreleg emergence (FLE) and time to complete tail resorption (TR), snout-vent length (SVL) and body weight (BW) at metamorphosis, mortality, gonadal development, thymus histology, disease rates, and the interaction between time to TR and SVL and BW at metamorphosis. Yet, according to LeBlanc and Wang, we simply

assessed the effects of nine pesticides individually (at 0.10 ppb) and in combination (each at 0.10 ppb) on time to foreleg emergence and time to complete tail resorption.

LeBlanc and Wang used their simple model to show that the effects of one of the pesticide mixtures on developmental time are predictable from the nonsignificant effects of the individual pesticides. Although they predicted the effects of a single pesticide mixture on a single variable, can their model predict the effects of even the single pesticides (propiconazole, λ-cyhalothrin, and atrazine) on the interaction between development and growth (Figure 5; Hayes et al. 2006), when none of these compounds significantly affected development alone and only atrazine affected size alone? Can their model predict the effects of atrazine plus *S*-metolachlor on the relationship between development and size or explain why the “inert” ingredients in the commercial mixture (Bicep II magnum; Syngenta Crop Protection U.S., Research Triangle Park, NC) appear to reduce this effect? Most certainly, the 70% meningitis infection rate in the surviving animals exposed to the nine-compound mixture cannot be predicted from exposure to the single pesticides, where disease rates were zero. The effect on development was the only parameter that fit LeBlanc and Wang’s model and thus explains their reason for focusing on this single measure and ignoring the other nine parameters we measured.

In conclusion, the questions raised in our article (Hayes et al. 2006) can be answered only with empirical evidence obtained from appropriately designed and carefully conducted laboratory experiments, not by simplified models that ignore interactions between independent variables and relationships between dependent variables. Finally, and most important, our data clearly show that examining individual pesticides one at a time does not reveal the magnitude of effects of low-dose chronic exposure to pesticide mixtures and thus does not allow us to accurately assess their impacts on amphibians. Practically all of the chemicals we examined had no significant effects alone, but this was certainly not the case when they were combined. Whether or not these interactions are response additive, concentration additive, or synergistic is irrelevant to the real question: Are we underestimating the impact? If we continue to base assessments on examinations of single compounds, the answer is “yes.”
